# Linking nutritional quality and climate impact of dairy and plant-based milk and yogurt alternatives: Evidence from three countries

**DOI:** 10.1016/j.crfs.2026.101598

**Published:** 2026-10-13

**Authors:** Marta Bianchi, Viktor Lundmark, Sandra Barman, Elinor Hallström, Susanne Bryngelsson

**Affiliations:** Department of Food Research and Innovation, RISE Research Institutes of Sweden, Frans Perssons väg 6, Gothenburg, | Box 857, 501 15, Borås, Sweden

**Keywords:** Dairy, Plant-based dairy alternatives, Nutritional life cycle assessment, Nutrition, Fortification, Climate, Functional unit

## Abstract

Dairy products are central to global diets, but their production contributes substantially to greenhouse gas emissions. Plant-based dairy alternatives (PBDAs) are increasingly consumed, yet assessments of their nutritional quality and environmental impacts remain limited. This study compared nutritional quality and climate impacts of milk, yoghurt, plant-based milk (PBMAs), and yoghurt (PBYAs) alternatives from France, Sweden, and the U.S. Nutritional quality was evaluated using Nutrient Density Score (NDS) and a modified Dietary Shift Index (DSI). Using a nutritional life cycle assessment approach, carbon footprints were expressed per 100 g, 100 kcal, and unit of nutritional quality.

Nutritional quality varied substantially across countries and was closely associated with fortification practices: fortification prevalence was highest in Sweden (76%) and lowest in France (16%). In France, milk outperformed unsweetened PBMAs (p = 0.0042, DSI), and sweetened yoghurt exceeded sweetened PBYAs (p = 0.0483, NDS; p < 0.001, DSI). Sweetened PBMAs had the lowest carbon footprint per nutritional unit (0.004–0.006 kg CO_2_-eq/DSI or NDS), while sweetened yoghurt was highest (0.020–0.037 kg CO_2_-eq). Using NDS and DSI as functional units increased the relative carbon footprint of dairy and PBDAs, aligning them to nutrient-dense animal foods, such as salmon and game, though DSI ranked milk and PBDAs more favorably than NDS.

These results suggest that functional units based on nutritional quality, particularly accounting for nutrients at risk in dietary transitions, provide a meaningful framework to evaluate sustainability. The study highlights the role of fortification and the need for further research on its nutritional and climate impacts in plant-based food alternatives.

## Highlights

• Nutritional quality of dairy and PBDAs varies across countries and reflects fortification.
• Milk and yoghurt outperform PBDAs in France, but not in Sweden or the U.S.
• PBDAs have lower carbon footprints than dairy per 100 g and per kcal.
• Food rankings change when nutritional quality is used as a functional unit.
• DSI yields more favorable rankings for milk and several PBDAs than the NDS.

## Graphical abstract

**Figure:**
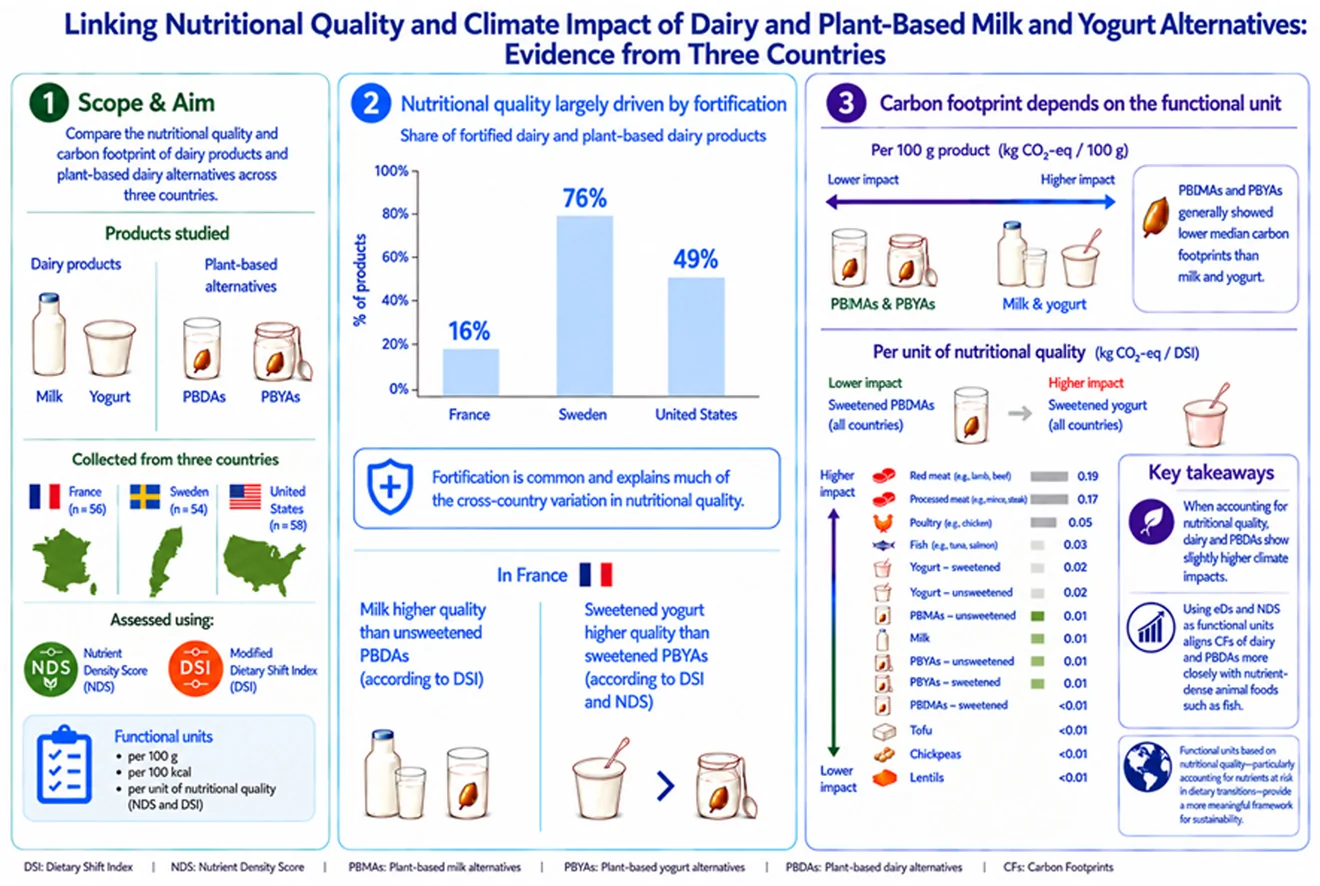

## Abbreviations

DRVs: Dietary reference values
DSI: Dietary shift index
LCA: life cycle assessment
MRIs Maximum recommended intakes
NDS: Nutrient density score
nFU: nutritional functional unit
PBDA: plant-based dairy alternatives
PBMA: plant-based milk alternatives
PBYA: plant-based yoghurt alternatives

## 1 Introduction

Dairy has a central role in the diet of numerous countries, with India leading globally in cow's milk consumption per capita and Denmark leading in Europe (Statista, 2024). However, dairy production, with differences across product types, together with red meat, accounts for a substantial share of the food system's total greenhouse gas emissions (GHGEs) (Poore and Nemecek, 2018; Meltzer et al., 2024). In affluent countries like Sweden, dairy products are estimated to contribute 23-30% of total dietary GHGE in adults (Moberg et al., 2020; Hallström et al., 2021).

Nevertheless, the nutritional role of dairy products in a healthy diet is supported by dietary guidelines globally, based on the natural content of high-quality protein, vitamin B2, and vitamin D (when fortified), as well as its important contribution to intakes of minerals such as calcium and iodine (Comerford et al., 2021; Bjørklund Holven and Sonestedt, 2024). Dairy products contain high-quality protein providing all essential amino acids and highly bioavailable minerals and vitamins, while nutritional profiles can vary across plant-based protein sources depending on factors such as protein content and fortification (White and Gleason, 2023). Accumulating evidence indicates that health effects associated with dairy consumption extend beyond their isolated nutrient composition and are attributable to the dairy food matrix, thereby fueling ongoing debate about whether and how dairy should retain a defined role in the so-called protein transition (Witard et al., 2025).

To acknowledge the nutritional role of dairy in the diet, while considering its environmental impacts, the Nordic nutrition recommendations emphasize the importance of a moderate consumption of dairy products, namely 350–500 ml of low-fat milk products daily, and suggest the inclusion of fortified plant-based alternatives if milk consumption is low (Blomhoff et al., 2023). The role of fermented dairy products, such as yoghurt, is particularly acknowledged (Tremblay et al., 2026). The Planetary Health Diet suggests that milk and derived dairy products take the largest share of animal sourced foods, recommending an average of 250 g per day (Rockström et al., 2025).

A nutritional life cycle assessment (nLCA) is a recommended approach to relate the environmental impacts of foods to their nutritional value (McLaren, 2021). This methodology builds on the principle that in order to be sustainable, a food product must provide adequate nutrition and a low environmental impact (Kyttä et al., 2025).

In this methodology, the use of a nutrient-based functional unit (nFU) is a common and valid practice (McLaren, 2021), expressing the environmental performance of foods in relation to single- or multi-nutrient-based FUs. For example, Vieux et al. identified relevant single-nutrient FUs for dairy as protein, vitamin A, folate, vitamin B12 and calcium, based on French food consumption data (Vieux et al., 2025). Most studies, however, compare the environmental sustainability of foods relative to nFUs based on multi-nutrient nutritional quality indicators rather than single-nutrient-based FUs (Aidoo et al., 2023; Katz-Rosene et al., 2023; Cardinaals et al., 2024; van Eijsden et al., 2025). Finnish researchers have recently proposed distinct nFUs for dairy products and plant-based milk alternatives (PBMAs), drawing on composite indicators that include nutrients for which dairy and PBMAs are among the leading dietary sources in Finland (Kovanen et al., 2024). Such product-group-specific nFUs may represent suitable tools to assess the nutritional implications of replacing milk with plant-based alternatives, particularly in contexts where differences in fortification practices influence nutrient composition.

However, when assessing the role of dairy in a sustainable diet more broadly and across a wide range of foods, a nutritional quality indicator based on a larger number of nutrients might be best suited as nFU. For such assessments, indicators such as the Nutrient Density Score (Vieux et al., 2025), the Nutrient Balance Concept (Fern et al., 2015) or the Qualifying Index (van Eijsden et al., 2025), all based on more than twenty different nutrients, have been proposed as useful. In certain studies, where foods are compared for their sustainability performance in the context of specific dietary requirements, the selection of nutrients to be included is driven by the nutritional and health needs of a specific population or by the risk for nutrient deficiency (Katz-Rosene et al., 2023; Ortenzi et al., 2025).

A further approach is based on the premise that dairy products should be regarded primarily as a source of dietary protein (Drewnowski, 2025). From this perspective, protein-focused nutritional metrics are particularly suitable for application in nLCA. Examples of such indicators include the NR-FIprot (Kyttä et al., 2023), the NRFprotein-sub score (Green et al., 2021) or the NRprot7 (Herrmann et al., 2024). When comparing the sustainability of protein sources, product-group-specific nFUs are best suited to highlight the nutritional consequences of changes in food intake in the context of dietary shifts, and their development has been identified as one of the topics for future research within the nLCA methodology (McLaren, 2021; Kyttä et al., 2023; Kyttä et al., 2023, 2023). The Dietary Shift Index, for example, includes six nutrients (protein, calcium, zinc, vitamins B2, B12 and D) estimated to be significantly lower as food consumption changes toward more sustainable plant-based diets (Kyttä et al., 2023).

Although research on nFUs in LCA is growing rapidly, relatively few studies have evaluated the environmental impact and nutritional properties of dairy and plant-based dairy alternatives (PBDAs) in parallel. Furthermore, most studies have so far focused on milk and plant-based alternatives to milk (Kovanen et al., 2024; Maree et al., 2024; Nowson et al., 2025). There is, therefore, a clear need to improve understanding of how different nFUs influence the combined assessment of dairy and PBDAs and to extend this knowledge to food groups beyond milk and PBMAs.

To address these knowledge gaps, we aimed to explore the implications of integrating nutritional quality into climate impact assessments of milk, yoghurt, PBMAs and plant-based alternatives to yoghurt (PBYAs).

The objectives of this study were to investigate the nutritional quality of dairy and PBDAs from three countries, to evaluate the performance of selected nutritional indicators, and to compare carbon footprints when nutritional indicators were used as nFUs. We applied the Nutrient Density Score (NDS), a nutritional indicator suitable for a broad comparison across food groups, and the Dietary Shift Index (DSI), a protein-targeted indicator suitable for dietary changes, to products from France, Sweden, and the U.S. In addition, dairy products and PBDAs were evaluated alongside a selection of other protein-rich foods. The purpose of this comparison was not to imply direct dietary substitutability between these foods, but rather to examine how different nutritional functional units influence food rankings when sustainability assessments are extended beyond dairy products alone.

The results will contribute to our knowledge about the large variation in nutritional quality of milk, yoghurt and their plant-based counterparts across countries, stimulating a reflection on the role of dairy and PBDAs in sustainable diets and on the impact of fortification practices and policies. Findings will further stimulate discussion and guide the selection of nutrient-based FUs in LCA studies of dairy and PBDAs.

## 2 Materials and methods

### 2.1 Included foods

This study included milk, yoghurt, PBMAs and PBYAs from three different national food composition databases: the French Food Composition Tables (French Agency for Food, Environmental and Occupational Health & Safety, 2025), the Swedish Food Composition Database (The Swedish Food Agency, 2024), the Food and Nutrient Database for Dietary Studies by the USDA (US Department of Agriculture, 2023).

The following related product typologies were excluded due to the heterogeneity of their composition and consumption habits: milk-based beverages, including flavoured milks (e.g., chocolate-flavoured milkshake); milk-based desserts; powdered or evaporated milk; condensed milk; milk and yoghurts from other animals than cow; quark. Unlike flavoured milk-based beverages, flavoured yoghurts, flavoured PBMAs and PBYAs were included. This resulted in 54 foods selected for Sweden (5 milks; 28 yogurts; 14 PBMAs and 7 PBYAs), 56 for France (10 milks; 26 yogurts; 11 PBMAsand 6 PBYAs), and 58 for the U.S (12 milks; 32 yogurts; 11 PBMAs and 3 PBYAs).

Dairy foods and PBDAs were classified in sub-categories based on the absence or presence of added sugars, flavours or other ingredients (e.g., fruit, cereals, chocolate) (Table 1). When a food item was indicated as a pooled average of both unsweetened and sweetened products, it was classified as sweetened (e.g., a PBMA product in the U.S. database was reported as a weighted average based on 80% unsweetened and 20% sweetened drinks).

**Table 1:** Milk, yoghurt and PBDAs classification in categories.

Food category	Foods included
Milk	Plain liquid milk, with varying fat content.
Yoghurt, unsweetened	Plain drinkable or spoonable yoghurt products, with varying fat content, including Greek yoghurt and kefir.
Yoghurt, sweetened or flavoured	Sweetened or flavoured drinkable or spoonable yoghurt products, with varying fat content, including Greek yoghurt, and yoghurts with added fruit, cereals or chocolate.
PB milk alternative, unsweetened	Plain plant-based beverages (e.g., oat, soy, almond), with varying fat content.
PB milk alternative, sweetened or flavoured	Sweetened or flavoured plant-based beverages (e.g., oat, soy, almond), with varying fat content.
PB yoghurt alternative, unsweetened	Plain plant-based yoghurt alternatives (e.g., soy, oat, coconut), with varying fat content.
PB yoghurt alternative, sweetened or flavoured	Sweetened or flavoured plant-based yoghurt alternatives (e.g., soy, oat, coconut), with varying fat content.

Additionally, 20 animal-based and plant-based protein-rich foods other than dairy and PBDAs were selected from the Swedish food database and included as reference foods. The selection was made to cover a variety of protein-rich food types (e.g., meat, fish, eggs, and legumes) rather than reflect population consumption patterns or dietary substitution scenarios. These foods were included exclusively to examine how the nutritional quality indicators and corresponding nutritional functional units influence the ranking of foods in sustainability assessments across different protein-rich food categories.

### 2.2 Nutrient composition data

The nutrient composition of the included foods was obtained from the country-specific food composition tables and refers to prepared foods, ready for consumption (French Agency for Food, Environmental and Occupational Health & Safety, 2025; The Swedish Food Agency, 2024; US Department of Agriculture, 2023). Available information concerning ingredients, fortification, and presence of non-nutritive sweeteners was also collected. The French database presented incomplete nutrient profiles in a few cases, which were then complemented based on the assumptions detailed in Supplementary 1.

### 2.3 Nutrient quality indicators

From the literature, we selected two indicators of nutritional quality based on nutrients with positive health effects—the Nutrient Density Score (NDS) (Vieux et al., 2025) and a modified Dietary Shift Index (DSI) (Kyttä et al., 2023)—as well as an indicator based on nutrients to limit, the Limiting Nutrient Index (LIM) (Vieux et al., 2025). The NDS is generally used for broad comparisons across food groups, whereas the DSI is a protein-targeted indicator developed for studies focusing on dietary changes.

These nutritional quality indicators assess the nutritional adequacy of the analyzed foods relative to recommended nutrient intakes and are expressed as a percentage of the dietary reference values (DRVs) or the maximum recommended intakes (MRIs) for LIM. Country-specific recommended energy intake and DRVs for age groups between 1 and 70 years were obtained from national authorities, as detailed in Supplementary 2.

The nutritional indicators were first calculated separately for each age and sex group. Subsequently, population-weighted averages were calculated based on demographic data for the three countries for the year 2024 (INSEE, 2025; Statistics Sweden, 2025; US Census Bureau, 2025) (Supplementary 3).

#### 2.3.1 Nutrient Density Score (NDS)

The NDS index was calculated as the arithmetic average of 23 nutrients positive to health (protein, fiber, linoleic acid, alpha linolenic acid, EPA + DHA, vitamins A, C, D, E, B1, B2, B3, B6, B9, B12, calcium, iron, potassium, magnesium, iodine, selenium, copper and zinc) (Supplementary Table 1), according to the following equation, based on (Vieux et al., 2025):

(Eq. 1) $$NDS=\frac{\sum_{i=1}^{i=23}Nutrient\;i/DRV\;i}{23}*100$$

where Nutrient_i_ is the amount (in g, mg, or *μ*g) of nutrient *i* provided by 100 kcal of each food and DRV_i_ are the country-, age-, and sex-specific recommended intakes as detailed in Supplementary 2. For the Swedish and U.S. products, NDS was calculated based on 22 nutrients, as the corresponding country-specific databases do not provide the content of copper and iodine, respectively.

#### 2.3.2 Modified Dietary Shift Index (DSI)

The DSI index was calculated as an arithmetic average of seven nutrients considered positive to health (protein, vitamins D, B2, B12, calcium, iron, zinc) (Supplementary Table 1), according to the following equation, based on (Kyttä et al., 2023):

(Eq. 2) $$DSI=\frac{\sum_{i=1}^{i=7}Nutrient\;i/DRV\;i}{7}*100$$

where Nutrient_i_ is the amount (in g, mg, or *μ*g) of nutrient *i* provided by 100 kcal of each food and DRV_i_ are the country-, age-, and sex-specific recommended intakes as detailed in Supplementary 2. Unlike in Kyttä et al. where DSI was calculated per 100 g, in the present study the index was calculated for a reference amount of 100 kcal to ensure consistency with the NDS and in accordance with the approach recommended by Bianchi et al. (2020). To better reflect potential changes in nutrient intake associated with dietary shifts towards more plant-based diets, the DSI was further modified in this study to include iron.

### 2.4 Limiting Nutrients Index (LIM)

The LIM index was calculated as the arithmetic average of three nutrients (saturated fat, total sugars, sodium), according to the following equation, based on (Vieux et al., 2025):

(Eq. 5) $$\mathrm{LIM}=\frac{\sum_{i=1}^{i=3}Nutrient\;i/MRI\;i}{3}*100$$

where Nutrient_i_ is the quantity (in g, mg, or *μ*g) of nutrient *i* provided by 100 g of each food and MRI_i_ are the country-, age-, and sex-specific maximum recommended intakes, as described in Supplementary 2. Total sugars were used instead of added sugars because food composition tables for France and the U.S. do not report added sugars values. Higher LIM values indicate a less favorable nutritional profile.

### 2.5 Carbon footprints

Carbon footprints for dairy, PBDAs and additional protein-rich foods were obtained from version 3.2 of the French database Agribalyse (Cornelus et al., 2025), which provides a matching at food item level with the French food composition tables (French Agency for Food, Environmental and Occupational Health & Safety, 2025). These data follow the recommended framework for LCA methodology as specified in ISO standards 14040 and 14044 (ISO 14044:2006; ISO 14040:2006).

Carbon footprints are based on life cycle inventory data encompassing system boundaries from cradle to factory gate, excluding packaging. For the reference foods, the system boundary included eventual weight change from cooking (e.g., lentils’ water absorption during boiling). The global warming potential (GWP) impact category was used to calculate the carbon dioxide equivalents for each food item, applying weighting factors from the IPCC 2021 AR6 (Masson-Delmotte, 2021).

For dairy and PBDAs, 22 distinct carbon footprints were retrieved and matched to 54 products (10 milks, 26 yoghurts and 18 PBDAs) from the French dataset. When direct matching was not available, a proxy from Agribalyse was applied. For three foods (i.e., a chestnut-based drink, a rice-based drink, and game) that lacked both a match and a proxy in Agribalyse, carbon footprints were retrieved from other sources ( RISE food climate database, 2025; Masson-Delmotte, 2021; Fiala et al., 2020).

Carbon footprints from Agribalyse and other sources were subsequently also matched with Swedish and U.S. nutritional datasets. Details are provided in Supplementary 4.

Carbon footprints of foods assessed were expressed relative to four different functional units: per 100 g of products (CO2e/100 g), per 100 kcal (CO2e/100 Kcal), and per NDS (CO2e/1-unit NDS) and DSI (CO2e/1-unit DSI).

### 2.6 Statistical analysis

Tests of normality indicated significant deviations from a normal distribution in all data subsets; therefore, nonparametric methods were used for all analyses (Hollander et al., 2015). Differences between product categories were assessed using Kruskal–Wallis tests, performed separately for each combination of country and indicator. When a significant overall difference was detected, post-hoc pairwise comparisons were conducted using Wilcoxon rank-sum tests with Bonferroni correction. Differences between milk (or PBMAs) and yoghurt (or PBYAs) are not considered relevant as these product typologies are intrinsically very different nutritionally. Data interpretation was therefore based on significant differences observed between a dairy product category and its PB counterpart, or between an unsweetened product category and its sweetened counterpart.

Additionally, differences between fortified and non-fortified products were assessed using a stratified Wilcoxon rank sum test, stratifying by country, with Bonferroni correction for multiple testing. The statistical analysis was performed using the R package *coin* (Hothorn et al., 2008).

### 2.7 Sensitivity analysis

Several additional assessments were conducted as part of a sensitivity analysis. The nutritional quality indicators NR-FI_milk_ and NR-FI_milk + PBD_ (Kovanen et al., 2024) were applied to dairy and PBDAs to evaluate whether a dairy- or PBMA-specific nutrient metric would provide different results than NDS and DSI. These indicators were not included in the main results because the primary analyses focused on nutritional quality indicators with broader applicability across food groups and dietary contexts. While the NR-FI_milk + PBD_ index can be applied to both dairy and plant-based dairy alternatives, the NR-FI indicators are developed based on the nutrients contributed by these products in the Finnish diet and were therefore included as part of the sensitivity analyses rather than as primary outcome measures.

In line with previous research (Bianchi et al., 2020; Vieux et al., 2025), a reference amount of 100 kcal was used for the calculation of nutritional quality. However, to assess whether 100 kcal was the most appropriate reference amount to compare dairy and PBDAs, NDS and DSI were additionally calculated per 100 g of product.

In this study, the content of nutrients to limit was assessed as a separate index (LIM). To evaluate the impact of LIM on the overall nutritional quality of dairy and PBDAs, two additional indicators were calculated by subtracting LIM from NDS and DSI (i.e., NDS-LIM, DSI-LIM) per 100 g of product.

A further sensitivity analysis was conducted by applying capping, whereby nutrient contents per 100 kcal of product were rounded to 100% of the country-, age-, and sex-specific DRVs when the calculated values exceeded the respective DRVs.

## 3 Results

### 3.1 Nutritional quality across dairy and PBDA categories

The nutritional quality of dairy and PBDA products across the three countries was assessed using the indicators NDS and DSI. The highest median nutritional quality was observed for unsweetened PBMAs in the U.S. according to both NDS (16.5) and DSI (22.0) (Table 2). The lowest median nutritional quality was observed among French products, specifically for sweetened or flavoured PBYAs according to NDS (2.8) and in unsweetened PBMAs according to DSI (2.4) (Table 2).

**Table 2:** Number of products, number of fortified products, median (MIN, MAX) of nutritional quality (NDS and LIM) and limiting nutrients (LIM) for French, Swedish and U.S. dairy and PBDAs.

Food grouping	N products			N fortified products			NDS (100 kcal)			DSI (100 kcal)			LIM (100 g)		
	Fr	Swe	US	Fr	Swe	US	Fr	Swe	US	Fr	Swe	US	Fr	Swe	US
Milk	10	5	12	2	4	10	9.32 (2.32,26.65)	9.65 (5.96, 12.79)	10.06 (7.38, 14.52)	16.84 (6.91, 57.45)	17.93 (9.62, 24.24)	20.41 (15.37, 30.78)	4.59 (3.53, 6.11)	4.84 (3.52, 7.12)	4.95 (3.56, 7.76)
PBMA unsweetened	7	10	3	1	7	0	3.89 (0.52, 8.06)	8.58 (3.86, 26.86)	16.52 (10.17, 26.64)	2.37 (0.36, 11.07)	11.81 (3.75, 24.82)	21.99 (19.84, 45.78)	3.76 (0.68, 6.30)	1.93 (0.97, 3.08)	1.28 (1.07, 3.71)
PBMA sweetened or flavoured	4	4	8	2	4	2	3.74 (0.46, 7.19)	10.41 (6.72, 14.71)	10.64 (9.03, 19.85)	4.18 (1.40, 7.70)	18.98 (13.82, 27.82)	20.06 (12.28, 34.18)	4.36 (2.76, 5.33)	3.85 (2.35, 5.74)	3.48 (1.76, 5.32)
Yoghurt, unsweetened	7	14	8	0	12	3	8.75 (2.03, 12.87)	7.75 (5.03, 11.35)	9.85 (6.55, 11.56)	14.82 (4.37, 23.04)	13.95 (6.90, 19.73)	21.64 (13.70, 26.32)	4.40 (2.36, 10.10)	5.46 (2.94, 9.95)	5.54 (2.63, 6.56)
Yoghurt, sweetened or flavoured	19	14	24	0	7	13	4.47 (2.19, 12.92)	5.36 (2.99, 7.98)	6.64 (4.59, 10.95)	8.08 (4.43, 19.95)	9.14 (4.17, 14.15)	14.63 (9.14, 24.93)	10.20 (3.04, 13.10)	8.37 (5.28, 15.30)	7.71 (5.00, 9.76)
PBYA unsweetened	3	2	0	1	2	0	7.83 (5.93, 8.17)	5.52 (4.39, 6.65)	-	9.10 (5.65, 9.99)	7.70 (4.11, 11.29)	-	1.22 (0.90, 2.28)	3.47 (3.21, 3.73)	-
PBYA, sweetened or flavoured	6	5	3	3	5	3	2.83 (0.87, 3.63)	5.36 (4.40, 5.62)	6.54 (4.27, 8.37)	3.37 (1.48, 5.20)	10.37 (9.85, 10.58)	8.22 (8.03, 10.65)	8.78 (7.53, 13.90)	5.33 (4.66, 5.64)	6.05 (5.71, 12.6)

The nutritional quality values reported in this section reflect the composition of products as recorded in the food composition databases. As products within each category differed substantially in ingredients, nutrient profiles, and fortification practices, the category-level results should be interpreted in the context of this within-category variation rather than as effects of individual product characteristics. The specific role of fortification is examined separately in Section 3.3.

Both NDS and DSI were able to significantly discriminate the nutritional quality across all assessed product categories in the French, Swedish and U.S. datasets (i.e., Kruskal-Wallis p-values ≤0,05) (Fig. 1). However, when pair-wise comparisons were performed using the Wilcoxon test, differences between product categories varied between the three datasets. In total, five relevant pair-wise comparisons showed statistical significance.

**Fig. 1:**
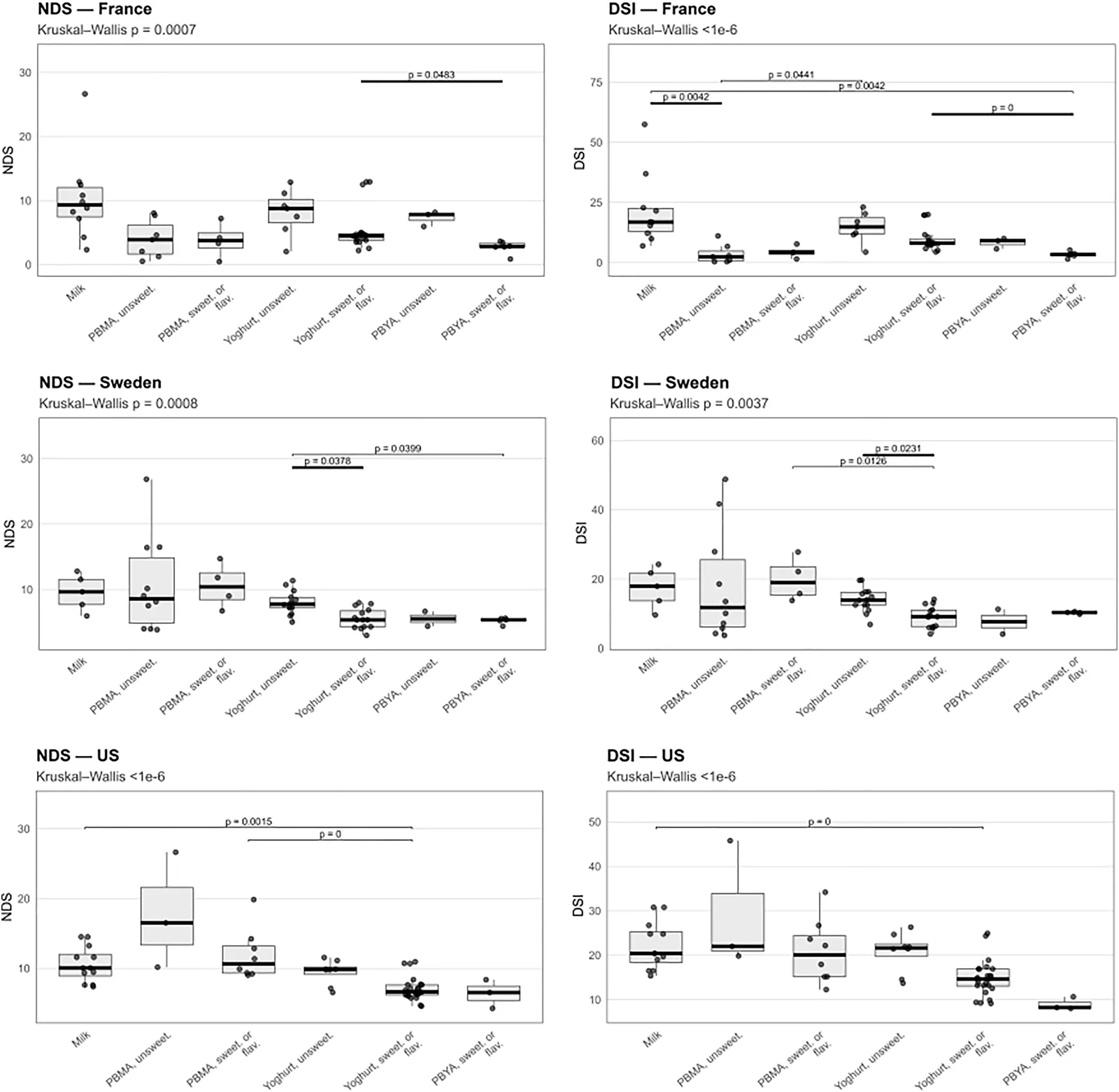
Population-weighted median values of nutritional quality in French, Swedish, U.S. products according to two indicators per 100 kcal. Overall differences between groups were assessed using the Kruskal–Wallis test. Significant pairwise differences from Bonferroni-adjusted Wilcoxon rank-sum tests are indicated by horizontal lines and corresponding p-values. Bold lines indicate nutritionally relevant significant differences. Results were considered statistically significant at p ≤ 0,05.

Regarding the quality of dairy vs. PBDAs, milk had significantly higher nutritional quality than unsweetened PBMAs according to DSI (p = 0.0042) in French products (Fig. 1). In contrast, neither NDS nor DSI indicated a significant difference between these two product categories in the Swedish and U.S. products. Nutritional quality of milk in these databases was generally similar to, or slightly lower, than that of PBMAs, although these differences did not reach statistical significance. Additionally, sweetened yoghurt had significantly higher nutritional quality than sweetened PBYAs according to both NDS (p = 0.0483) and DSI (p = 0) in French products.

Regarding differences between unsweetened and sweetened products, unsweetened dairy yoghurt had statistically higher nutritional quality than sweetened dairy yoghurt according to both NDS (p = 0.0378) and DSI (p = 0.0231) in Swedish products.

No statistically significant differences were observed among U.S. product categories either when comparing the nutritional quality of dairy and PBDAs, or for unsweetened vs. sweetened products.

These findings were further supported by a sensitivity analysis incorporating two dairy- and PBDA-specific indicators. Both NR-FI_milk_ and NR-FI_milk + PBD_ were statistically significant higher in milk compared to PBMAs in French products (p = 0.0252 and p = 0.0462, respectively). Moreover, these indicators effectively distinguished between sweetened and unsweetened dairy yoghurt, as well as between dairy yoghurt and PBYAs, although with differences in the three countries (Supplementary Fig. 1S).

Results from the sensitivity analysis examining the impact of expressing nutritional quality per 100 g versus per 100 kcal are presented in Supplementary Fig. 2S. Analyses based on 100 g revealed a greater number of statistically significant—yet nutritionally negligible—differences between French and U.S. products compared with analyses based on 100 kcal. In contrast, for Swedish products, pairwise comparisons showed no statistically significant differences between any product groups.

Notably, switching the reference amount from 100 kcal to 100 g did not affect DSI's ability to differentiate the nutritional quality of French products. In contrast, NDS results were influenced by this change: the nutritional quality of milk became significantly higher than that of unsweetened PBMAs, whereas sweetened yoghurt was no longer significantly different from sweetened PBYAs.

### 3.2 Content of limiting nutrients in dairy and PBDA categories

Among the analyzed foods, the lowest and highest LIM values per 100g were observed among French products. Specifically, unsweetened PBYAs had the lowest LIM (1.22), whereas sweetened or flavoured yoghurt showed the highest LIM value (10.20) (Table 2).

LIM values differed significantly across all assessed product categories in the French, Swedish and U.S. datasets (Fig. 2). In pair-wise comparisons of Swedish products, milk had a significantly higher LIM value than unsweetened PBMAs (p = 0.0252), unsweetened yoghurt had lower LIM than sweetened or flavoured yoghurt (p = 0.0105), and sweetened or flavoured yoghurt had a higher LIM than sweetened or flavoured PBYAs (p = 0.0168).

**Fig. 2:**
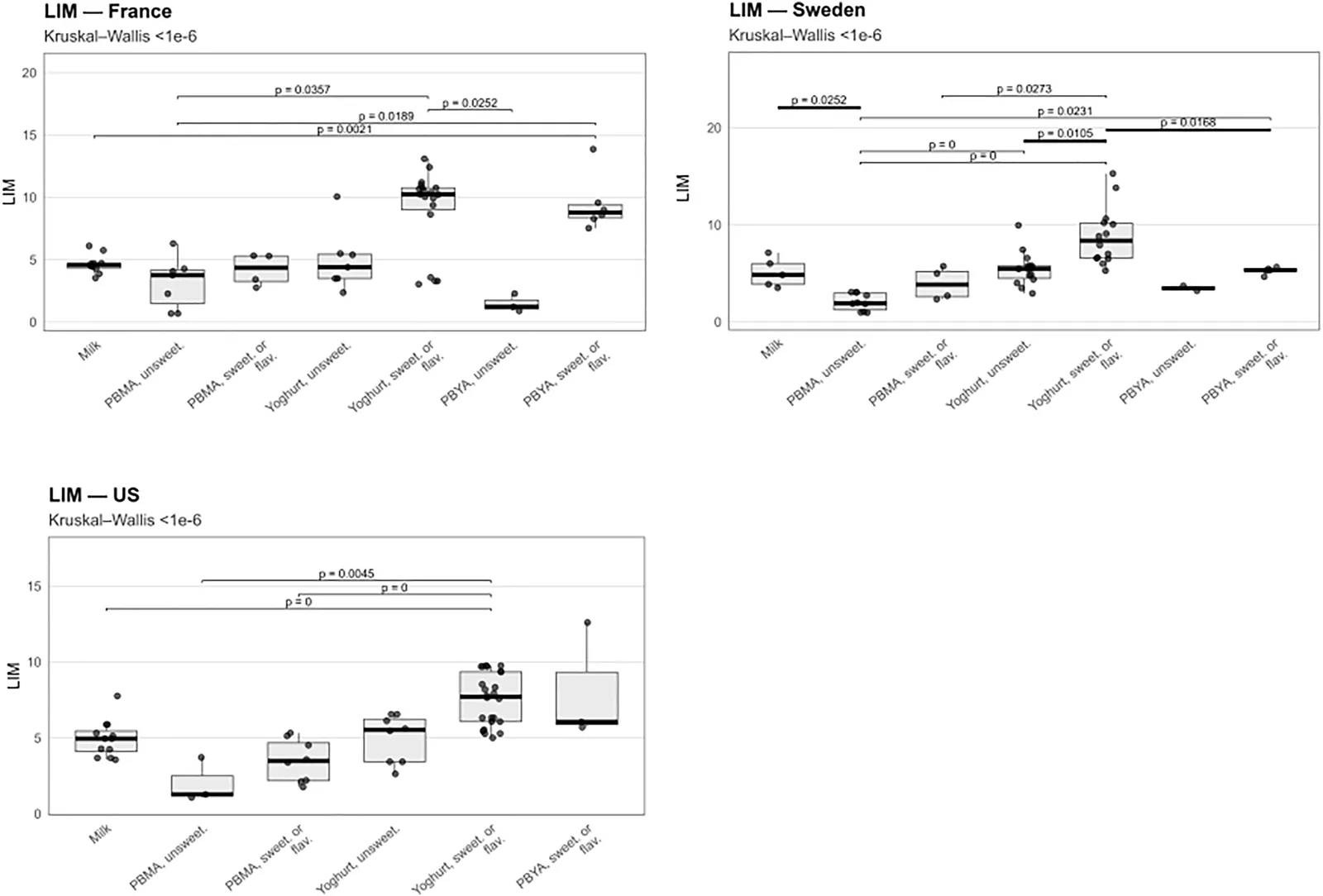
Population-weighted median LIM scores (saturated fat, total sugars, sodium) for dairy and PBDAs in France, Sweden, U.S. Nutrient content per 100 g of product. Overall differences between groups were assessed using the Kruskal-Wallis test. Significant pairwise differences from Bonferroni-adjusted Wilcoxon rank-sum tests are indicated by horizontal lines with corresponding p-values. Bold lines indicate nutritionally relevant significant differences. Results were considered statistically significant at p ≤ 0,05.

Results from the sensitivity analysis in which the LIM score was subtracted from both NDS and DSI are presented in Supplementary Fig. 3S. Accounting for nutrients to limit resulted in a significantly lower nutritional quality of sweetened or flavoured yoghurt compared with the corresponding PBYAs among Swedish products, for both NDS (p = 0.0391) and DSI (p = 0.0084), and among French products for DSI (p = 0.0151). Additionally, among French products, subtracting LIM from NDS and DSI led to a levelling of the median nutritional quality of milk and unsweetened PBMAs.

### 3.3 Fortification and overall nutritional quality

In the French dataset, only 16% of the assessed food products (nine out of 56) were reported as fortified. In contrast, the proportion of fortified products was substantially higher in the Swedish and U.S. databases, at 76% (41 out of 54) and 53% (31 out of 58), respectively (Table 2).

Vitamin D was the most commonly fortified nutrient in Swedish and U.S. products, followed by calcium and vitamin B12. In the U.S., milk products were also frequently fortified with vitamin A (Food and Drug Administration, Department of Health and Human Services, 2026). In fortified products, vitamin D content never exceeded the DRV for adults.

Among French products, riboflavin exceeded the DRV for adult men and women (18-70 yrs) in two products, both fortified milk products with 1,2% fat content. One of these products was fortified with multiple vitamins and contained 2,5 times the DRV for riboflavin per 100 kcal but did not exceed the reference value for any other nutrient.

Among Swedish products, two almond drinks exceeded the reference value for adult women (25-50 yrs) for vitamin E, and one of these also exceeded the DRV for calcium. A similar pattern was observed in U.S. products, where only vitamin E and calcium exceeded the DRV for adult women (31–50 yrs) in two and one almond-based products, respectively.

Across the three datasets, a few additional nutrients exceeded the DRV when NDS and DSI were calculated for children and adolescents.

When capping was applied (i.e., nutrient contents exceeding 100% of the relevant DRV per 100 kcal were truncated at 100% of the DRV), it only had a marginal effect on the relative nutritional performance of dairy and PBDAs across the three datasets, affecting only the differentiation between yoghurt and PBYAs (both sweetened) among French products according to NDS, which was no longer significant after capping (Supplementary Fig. 4S).

Across most product categories, the overall nutritional quality of fortified products tended to be higher than that of unfortified products, although this difference reached statistical significance only for milk when the mean of French, Swedish, and U.S. products was considered (Fig. 3).

**Fig. 3:**
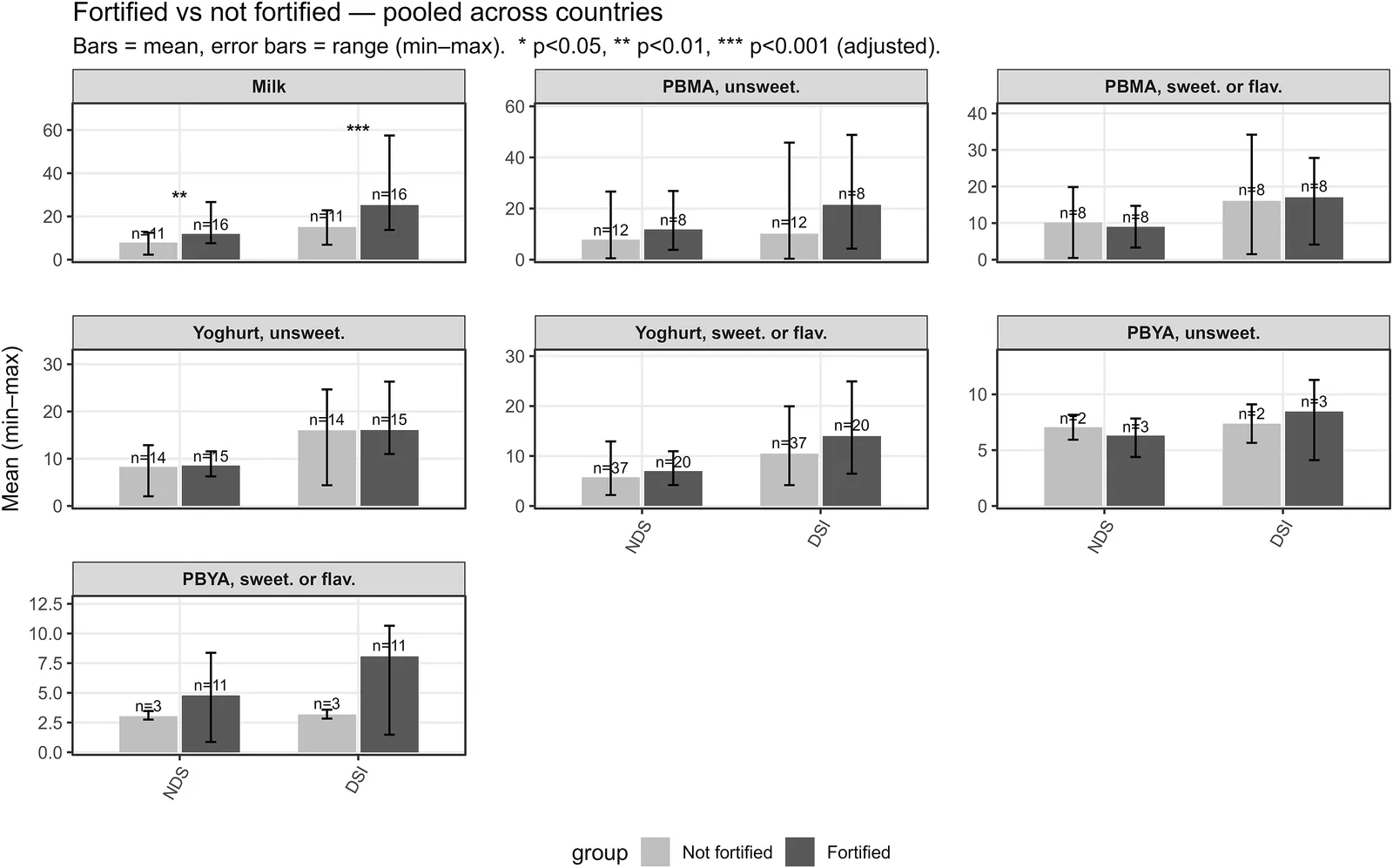
Nutritional quality according to NDS and DSI in fortified and not fortified products. Mean values for French, Swedish and U.S. dairy and PBDAs, per 100 kcal. Differences between fortified and not fortified products were assessed using stratified Wilcoxon rank sum tests with Bonferroni correction for multiple testing.

A more detailed examination of the country-specific datasets confirmed that fortified products generally exhibited higher nutritional quality than unfortified products among French and Swedish dairy and PBDAs. However, this difference was less pronounced across several product categories in the U.S. (Supplementary Fig. 5S–7S).

### 3.4 Food ranking based on nutritional quality

Fig. 4 illustrates a ranking of assessed dairy and PBDA products and reference foods based on their nutritional quality. According to the results, cod had the highest NDS value per 100 kcal (24.79) whereas the average of unsweetened PBYAs from France, Sweden and the U.S. had the lowest value (3.61). Using DSI, game meat exhibited the highest nutritional quality (25.77) and peanut butter the lowest (2.55).

**Fig. 4:**
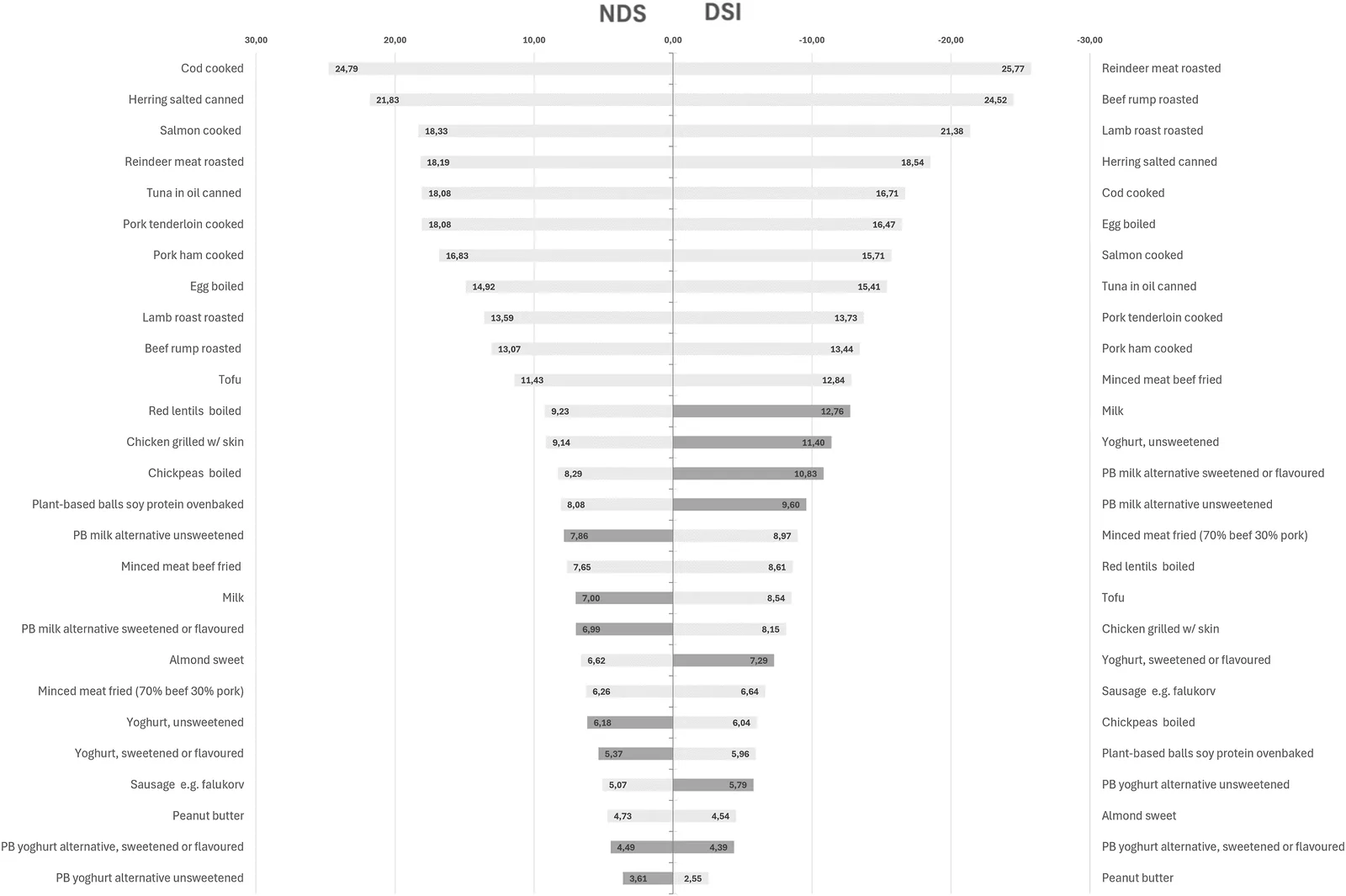
Ranking of nutritional quality (highest to lowest) according to NDS and DSI in dairy, PBDAs and 20 foods from other groups. Dark grey bars are average data for France, Sweden and the U.S. based on population weighted averages. Light grey bars are population-weighted nutritional quality from single foods from the Swedish Food Composition database. All data is expressed as nutritional quality per 100 kcal of products ready for consumption.

**Fig. 5:**
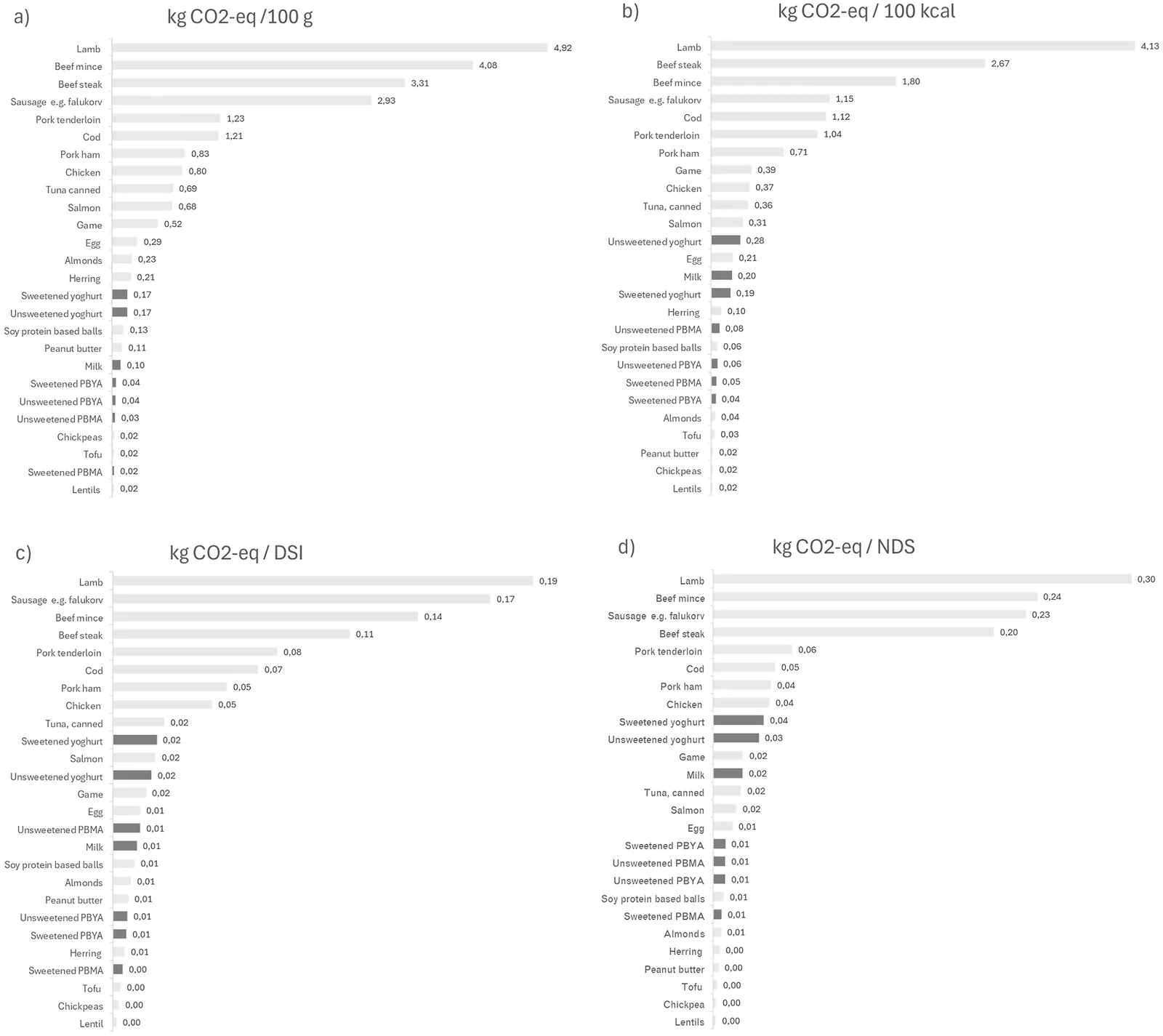
Carbon footprints of dairy, PBDAs and reference foods, expressed as kg CO2-eq per 100 g, 100 kcal, one unit of DSI and one unit of NDS. Dark grey bars are based on average nutritional quality for French, Swedish and the U.S. products. Light grey bars are based on nutritional quality from single foods from the Swedish Food Composition database. All climate data is obtained from Agribalyse.

**Fig. 6:**
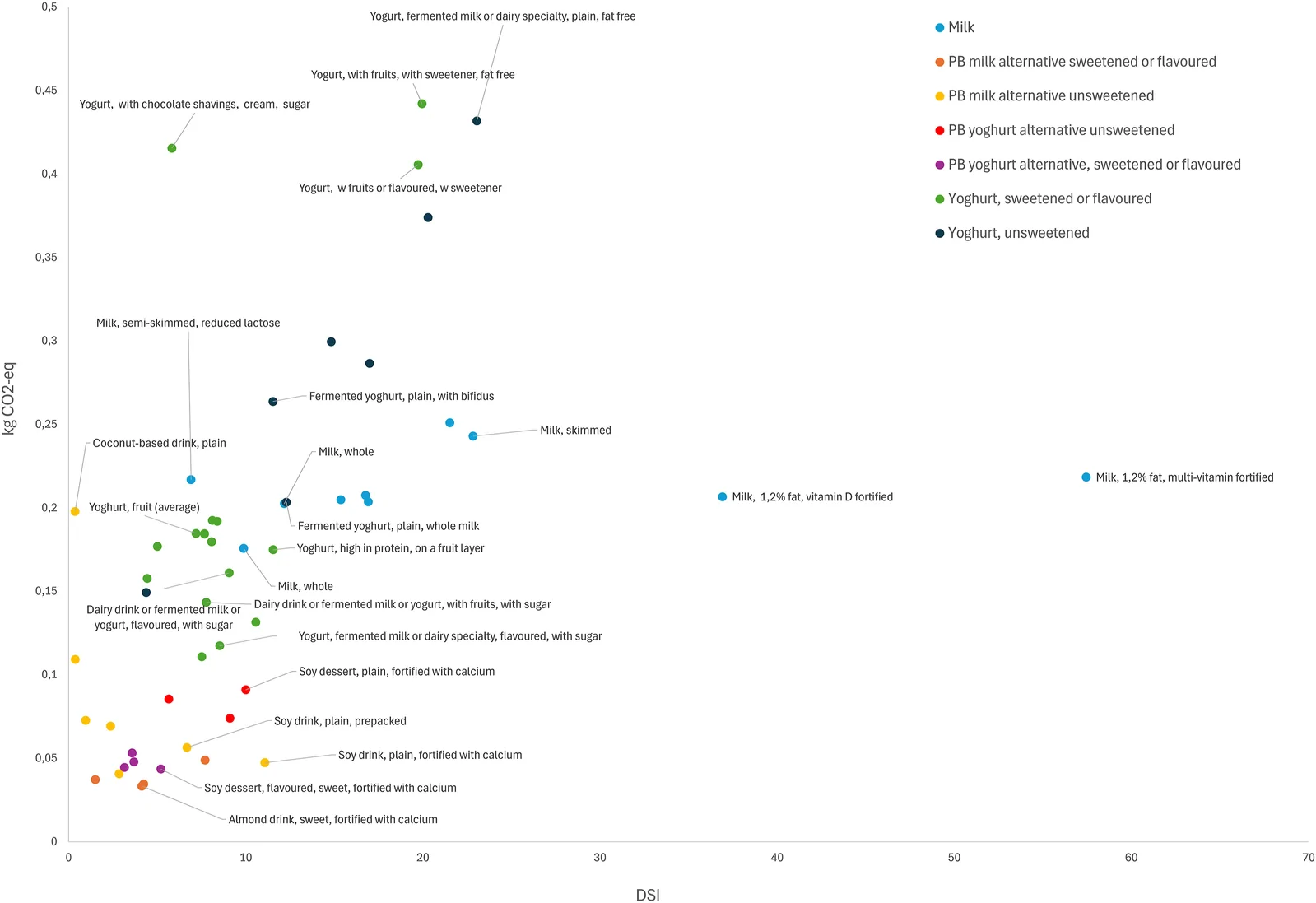
Climate impact for dairy and PBDA products from France and nutrient quality assessed by the Dietary Shift Index. Median values per 100 kcal of cooked/ready to eat food product at factory gate.

Overall, all fish products, most meat products, eggs and legumes had higher nutritional quality than dairy and PBDAs when assessed by NDS on a calorie basis. When the nutritional quality was estimated by DSI, chicken and legumes ranked lower, while most dairy and PBDA categories improved their relative position. Milk, unsweetened yoghurt, and both unsweetened and sweetened PBMAs ranked in the middle range and above chicken and legumes.

The ranking positions of milk, yoghurt and their PB counterparts relative to the protein-rich reference foods varied across the three datasets and are provided in Supplementary (Fig. 8S). Country level results confirmed that DSI gives a more favorable ranking of dairy and PBDAs compared with NDS, relative to the selected reference foods. Furthermore, dairy and PBDAs had the most favorable ranking in the U.S., followed by Sweden and France, according to the DSI.

The content of nutrients to limit in reference foods compared to dairy and PBDAs is shown in Supplementary (Fig. 9S). Per 100 g, canned herring had highest LIM value (9.76) and sweet almonds the lowest (1.03).

### 3.5 Carbon footprints and nutritional quality

Carbon footprint values for dairy and PBDAs (n = 22) ranged from 0.01 kg CO_2_-eq/100 g for almond drink to 0.51 kg CO_2_-eq/100 g for yoghurt with chocolate shavings (Supplementary, Fig. 10S). At the product category level, sweetened or flavoured PBMAs exhibited the lowest median carbon footprint (0.02 kg CO_2_-eq/100 g), whereas unsweetened yoghurt showed the highest (0.17 kg CO_2_-eq/100 g) (Fig. 5a).

When expressed per 100 kcal, the lowest and highest carbon footprints were observed for sweetened or flavoured PBYAs (0.04 kg CO_2_-eq/100 kcal) and for unsweetened yoghurt (0.28 kg CO_2_-eq/100 kcal), respectively (Fig. 5b).

When carbon footprints were expressed per the average nutritional quality of French, Swedish and U.S. products, the lowest value was observed for sweetened or flavoured PBMAs (0.004 kg CO_2_-eq/DSI and 0.006 kg CO_2_-eq/NDS), and the highest value for sweetened or flavoured yoghurt (0.020 kg CO_2_-eq/DSI and 0.037 kg CO_2_-eq/NDS) (Fig. 5c-d).

Fig. 6 illustrates the carbon footprints and nutritional quality (assessed using DSI) of French dairy and PBDA products, plotted on separate axes. Carbon footprints showed a relatively higher variation among yoghurts, both unsweetened and sweetened, which exhibited the highest climate impacts. Among milk products, carbon footprints clustered around intermediate values, but nutritional quality showed considerable variation, with the highest DSI scores observed in this group. PBMAs and PBYAs generally clustered at the lower end of both nutritional quality and climate impact ranges, except for a coconut-based beverage, which displayed an intermediate climate impact (0.05 kg CO_2_-eq/100 kcal). A similar pattern was observed when nutritional quality was assessed using the NDS (Supplementary Fig. 11S).

The median climate impacts of the seven dairy and PBDA categories were ranked alongside reference protein foods using four different functional units (Fig. 5). When expressed per 100 g, dairy and PBDAs ranked lower than all other animal-based protein sources. However, when calories were used as the functional unit, yogurt and milk exhibited climate impacts comparable to, or slightly higher than, those of eggs and herring. When carbon footprints were expressed per unit of nutritional quality, dairy and PBDAs shifted toward higher relative impacts, aligning with or exceeding those of other nutrient-dense animal products such as salmon, tuna, and game. Compared with the NDS, the use of the DSI as the functional unit resulted in more favorable rankings for milk and PBDAs. In contrast, plant-based protein sources such as lentils, tofu, and chickpeas consistently exhibited the lowest carbon footprints, regardless of the functional unit applied.

## 4 Discussion

This study explored the nutritional quality and climate impacts of milk, yoghurt and PBDAs using a nutritional LCA approach. It is well established that the extent of nutrient fortification in dairy products and PBDAs influences their overall nutritional quality and varies widely across countries due to differences in legislation and customary practices (Siegrist et al., 2024; Nowson et al., 2025). To account for this heterogenicity, products from two European countries (France and Sweden) and the United States were included in this assessment. Two nutritional indicators, previously applied to food sustainability assessments (i.e. the Nutrient Density Score (NDS) (Vieux et al., 2025), and the Dietary Shift Index (DSI) (Kyttä et al., 2023)), were evaluated for their ability to differentiate between unsweetened and sweetened or flavoured products, as well as between dairy products and their plant-based counterparts. These indicators were then applied as functional units for carbon footprint calculations.

Our results confirm that the nutritional quality of dairy products and PBDAs varies across countries and is influenced by fortification practices. In countries with low dairy and PBDAs fortification rates, such as France, milk and yoghurt contained relatively higher levels of health-promoting nutrients than their plant-based counterparts. In contrast, in Sweden and the U.S., where a substantial proportion of both dairy and PBDAs are fortified, nutritional quality was relatively homogeneous across product categories. Incorporating nutrients to be limited into the NDS and DSI indicators helped to better elucidate quality differences, particularly among Swedish products. Unsweetened products generally exhibited higher overall nutritional quality than sweetened or flavoured products in this study, with the most pronounced differences observed for dairy yogurt. When nutritional quality was used as nFU, dairy and PBDAs shifted towards relatively higher carbon footprints, aligning with or exceeding those of other nutrient-dense animal products such as salmon, tuna, and game. While PBDAs generally showed lower carbon footprints than dairy products, expressing climate impacts per unit of nutritional quality narrowed the differences between categories. Compared to NDS, DSI led to a more favorable ranking of carbon footprints for milk and several PBDAs relative to other protein-rich foods. However, given the uncertainty inherent in the limited number of carbon footprint values to represent diverse dairy and PBDA products, small differences in rankings should be interpreted with caution.

### 4.1 Performance of nutritional quality indicators

Both the NDS and the DSI indicators measure nutritional adequacy of a food relative to nutrient recommendations and are based on 23 and 8 nutrients positive to health, respectively (Vieux et al., 2025; Kyttä et al., 2023). Nutrients included in the DSI are selected based on the risk of suboptimal intake associated with dietary transitions toward more plant-centered diets, which may occur when dairy products are replaced with plant-based alternatives (Kyttä et al., 2023). We modified the original DSI algorithm by integrating iron, a nutrient with increased risk of deficiency in dietary transitions towards reduced meat consumption (Hallström et al., 2025).

Nutritional indicators should be able to rate food quality consistently with the dietary guidelines (Bianchi et al., 2020). In dairy and PBDAs one such aspect is the ability to discriminate between unsweetened and sweetened or flavoured products, which likely reflects the content of added sugars. Among Swedish products, both NDS and DSI showed lower nutritional quality in sweetened or flavoured yogurts compared with unsweetened yogurts. A similar trend was observed in products from the U.S., and generally in unsweetened vs. sweetened PBMAs, without reaching statistical significance. These findings suggest that, while both nutritional quality indicators generally classified sweetened products as lower in overall nutritional quality than their plain counterparts, the magnitude and detectability of this difference varied across countries. The fact that we did not observe a clearer quality difference between sweetened and unsweetened products can be partly explained by the limitation that NDS and DSI capture only protein, vitamins, and minerals, but not total or added sugars.

The inclusion of nutrients to limit (i.e., saturated fat, total sugars, and sodium) in a functional unit used in nutritional LCA has been widely debated (McLaren, 2021), and several approaches have been proposed to address related methodological challenges (Cortesi et al., 2024). One such approach suggests that the nutrients to limit should be accounted for in a separate score (Green et al., 2023). In this study we follow the approach of Vieux (Vieux et al., 2025), and present the content of total sugars, saturated fat and sodium per 100 g of product as a separate indicator (i.e., LIM). Total sugars were used in the LIM index due to unavailable data on added sugars in two of the three assessed databases. On a weight basis, LIM tended to be higher in yoghurt and PBYAs compared to milk and PBMAs. Additionally, among Swedish products, sweetened or flavoured yoghurt had significantly higher LIM than unsweetened yoghurt, and milk and yoghurt had higher content of limiting nutrients compared to their plant-based counterparts, explained by differences in sugars and saturated fat content, respectively. A previous study on PBDAs on the Swedish market also observed that flavoured PBYAs had higher energy and total sugars content compared to plain products (Moshtaghian et al., 2024).

As part of a sensitivity analysis, we assessed differences in nutritional quality across dairy and PBDA categories after subtracting LIM from the nutritional quality scores NDS and DSI. The results indicate that accounting for LIM can either attenuate or accentuate differences between product categories. For example, among French products—where milk had significantly higher nutritional quality than unsweetened PBMAs, due to a more limited fortification of the latter—subtracting LIM led to a levelling of their relative nutritional quality. In contrast, among Swedish products, where fortification is more widespread, incorporating LIM resulted in more pronounced differences, with sweetened yoghurt scoring lower than the unsweetened counterparts and dairy yoghurt scoring lower than corresponding PBYAs.

From a nutritional and health perspective, accounting for nutrients to limit in the NDS and DSI scores enables a more balanced comparison of foods. However, when these scores are applied in nLCA, it is recommended to avoid negative values in the functional unit, which may occur when nutrients to limit are subtracted (McLaren, 2021).

### 4.2 Nutritional quality of dairy and PBDAs across countries and the role of fortification

While dairy and PBDAs can both have a place in a healthy and sustainable diet, a direct comparison of their nutritional quality can help elucidate potential nutritional impacts in case of total substitution of dairy with PBDAs. Among French products, which are mainly not fortified, significant differences between dairy products and their plant-based counterparts consistently indicated higher nutritional quality for dairy, with significant differences observed using both NDS and DSI. These results remained largely unchanged when applying indicators specifically developed for nLCA of dairy and PBDAs (i.e. NR-FI_milk_ and NRF_milk + PBD_) (Kovanen et al., 2024), as part of a sensitivity analysis. Several studies have compared the nutrient profile of milk and PB alternatives in Europe, confirming that dairy milk generally provides higher nutrient density, more protein, but also higher content of saturated fat (Kovanen et al., 2024; Siegrist, 2024; Nowson et al., 2025), although the comparison is highly influenced by the degree of fortification of the plant-based alternatives.

Differences in nutritional quality observed in our study were consistent with the substantial differences in fortification practices across the three countries. In Sweden, both dairy and PBDAs were fortified to a greater extent (76% of assessed products) compared to the U.S. and France, reflecting mandatory vitamin D fortification in milk and PBDAs. Fifty-three percent of the assessed U.S. products were fortified, due to voluntary fortification of milk with vitamins A and D (Food and Drug Administration, Department of Health and Human Services, 2026). Among French products, only 16% were fortified (2 milk products and 7 PBDAs), on a voluntary basis, since no mandatory fortification is required in France. The low fortification rate of PBDAs in France is likely to be an important explanation for the lower nutritional quality observed for PBDAs relative to dairy products in this country. The large variation in fortification of dairy analogues across Europe, with France ranking lowest among the assessed countries, has also been demonstrated in previous studies (Siegrist et al., 2024). Despite substantial variation in fortification practices across countries, when average NDS and DSI values for French, Swedish, and U.S. products were considered, fortified products generally exhibited higher nutritional quality than their unfortified counterparts across most categories. The clearest effect was observed for milk, where fortified products had significantly higher NDS and DSI values than non-fortified products.

Fortified nutrients rarely exceeded the recommended nutrient requirements for adults, but this occurred more frequently when using intake recommendations for children, and then even applied to some nutrients naturally present in dairy (e.g., vitamin B2). However, when applying capping to nutrients exceeding their DRVs, the results remained largely unchanged, confirming the marginal effect of overestimating the nutritional quality of the analyzed foods, especially for the younger age groups, which are demographically less relevant in the population-weighted averages.

On a calorie basis, dairy and PBDAs on average had a lower nutritional quality than other animal- and plant-based protein sources, except high energy dense plant foods (e.g., peanut butter or almonds) or mixed-meat products which had lower quality, in line with what observed by other authors (van Eijsden et al., 2025). However, DSI resulted in a more favorable ranking of dairy and PBDAs than when NDS was used, likely due to its narrower and more targeted nutrients selection. Our findings suggest that the ranking of dairy and PBDAs relative to other foods is impacted by the choice of nutritional indicators. When comparing protein sources based on nutrients relevant to dietary transitions, dairy and PBDAs showed an intermediate nutritional quality.

### 4.3 Nutritional functional units and climate impact assessment

Beyond nutritional aspects, the replacement of dairy with PBDAs may contribute to a reduction in greenhouse gas emissions from the diet (Blomhoff et al., 2023). In line with previous findings (Kovanen et al., 2024), our results showed that PBMAs and PBYAs had lower median carbon footprints that milk and yoghurt, regardless of the chosen functional unit, except for the relative ranking of milk and unsweetened PBMAs when DSI was used as a functional unit.

Although PBMAs and PBYAs generally exhibited lower carbon footprints than milk and yoghurt, respectively, the magnitude of these differences was reduced when impacts were expressed per unit of nutritional quality rather than per mass or calorie. Nevertheless, PBDAs retained a climate advantage across most product categories. These findings should be interpreted in light of the uncertainties associated with the climate impact estimates, including variability in production systems and methodological assumptions underlying the original LCA studies, the use of a limited number of carbon footprint values to represent diverse dairy and PBDA products, and the exclusion of emissions associated with the production of fortified nutrients. Such uncertainties may affect the precise magnitude of estimated carbon footprints and the relative ranking of individual product categories but are unlikely to alter the overall pattern observed in this study.

The impact of the FU choice became more relevant when dairy and PBDAs were compared to other foods. When carbon footprints were expressed per calorie—and even more so per unit of nutritional quality—dairy and PBDAs shifted some positions toward higher relative climate impacts, compared to other protein sources. However, the bottom and the top of the food ranking remained largely unchanged, except for some high energy-dense foods (e.g., peanut butter and almonds) that shifted position across functional units. Milk and yoghurt showed carbon footprints per unit of DSI or NDS in line with those of other highly nutritious animal protein sources (e.g., salmon or game), while PBDAs retained an advantage in terms of lower relative carbon footprints and better ranking.

Although similar rankings were obtained when carbon footprints were expressed per nutritional quality, DSI was able to rank milk and several PBDA categories slightly more favorably than NDS. The DSI—characterized by a more restricted and tailored selection of nutrients—therefore appears better suited to highlight the nutritional quality of dairy and PBDAs relative to other protein sources.

### 4.4 Methodological considerations, strengths and limitations

A common limitation of nutritional quality indicators, including NDS and DSI, is that they account only for nutrient content, without distinguishing between food sources, chemical forms, or bioavailability within specific food matrices—factors known to differ substantially between animal- and plant-based foods and to influence overall nutritional quality (Fresán et al., 2026). Some attempts have been made to account for differences in iron and zinc bioavailability (Kyttä et al., 2023) or protein quality and digestibility (McAuliffe et al., 2023; Tukiainen et al., 2025) in nLCA. These aspects may be particularly relevant when comparing dairy products with PBDAs, as an increasing body of evidence suggests that the dairy food matrix plays a decisive role in shaping the health effects of dairy products by, for example, attenuating the expected negative health impacts of saturated fatty acids in fermented dairy foods such as yoghurt (Mulet-Cabero et al., 2024). Finally, more research is needed to fully explore the possibility to include bioavailability and other health-related factors into nutritional quality indicators for LCA. In this study, some nutrient data were missing from the databases, requiring several assumptions to fill these gaps. For example, iodine content was not available in the U.S. database and was therefore excluded from the NDS calculations for products from this country. This may have led to an underestimation of their overall nutritional quality, as iodine is a nutrient that can be abundant in dairy products (van der Reijden et al., 2017).

Further, the comparison between fortified and non-fortified products provides an overall assessment of differences according to fortification status. However, the contribution of individual fortified nutrients cannot be separated in this analysis, as both the nutrients used, and their levels vary across products.

A further limitation of this study is that environmental performance was assessed solely through CO_2_ emissions. Although climate impact is a key sustainability indicator, it does not capture other relevant environmental dimensions such as freshwater use, land occupation, biodiversity impacts, or eutrophication potential. Consequently, some environmental trade-offs between products may remain undetected. Additionally, a relatively small set of carbon footprint values was used to represent the wide variety of dairy and PBDA products, introducing uncertainty into the results. Furthermore, the carbon footprint estimates did not account for the climate impacts associated with the production of fortified nutrients. This limitation is consistent with most LCA studies of fortified products.

In this study, the inclusion of additional protein-rich foods should be interpreted as a methodological exercise rather than a comparison of directly interchangeable foods. Dairy products, PBDAs, fish, legumes, meat, nuts and eggs serve different roles within dietary patterns. These foods were included to explore how nutritional functional units influence sustainability rankings across diverse food categories, thereby providing insight into the broader applicability of the NDS and DSI frameworks.

In this work, we applied a nutritional LCA approach at the food level, placing greater emphasis on nutrients associated with positive health outcomes when comparing dairy, PBDAs, and other protein-rich foods. However, extending this approach to the dietary context necessarily requires a more comprehensive consideration of nutrients of public health concern (e.g., saturated fat, sugars, and sodium). Likewise, a broader sustainability assessment of dairy and PBDAs relative to other dietary protein sources should incorporate additional environmental dimensions, such as biodiversity loss and freshwater use.

## 5 Conclusions

Our results confirm that the nutritional quality of dairy products and PBDAs varies across countries and is closely associated with differences in fortification practice. When accounting for some nutrients with positive health effect, milk and yoghurt exhibited higher nutritional quality than their plant-based counterparts, but only among French products, which are largely unfortified. In contrast, in Sweden and the United States—where a substantial proportion of both dairy and PBDAs are fortified—nutritional quality was relatively similar across product categories. Including saturated fat, total sugars, and sodium (i.e., the LIM index) in nutritional quality assessments could alter the observed differences between product categories by either attenuating or accentuating them.

Overall, the DSI tended to rate the nutritional quality of dairy products and PBDAs more favorably than the NDS when expressed on a per-calorie basis, particularly in comparison to other protein-rich foods. PBMAs and PBYAs generally showed lower median carbon footprints than milk and yoghurt. However, when nutritional quality was used as the nFU, both dairy products and PBDAs shifted toward slighlty higher carbon footprints, aligning with or even exceeding those of other nutrient-dense animal products such as salmon, tuna, and game. Compared to the NDS, the DSI resulted in a more favorable ranking of carbon footprints for milk and several PBDAs relative to other protein-rich foods.

This study demonstrates that comparisons of carbon footprints between dairy, plant-based alternatives, and other protein sources become more insightful when based on functional units reflecting nutritional quality, particularly those capturing nutrients critical to dietary transitions. It also highlights the important role of fortification and underscores the need for further research on the nutritional and climate impact of fortification in plant-based alternatives to animal foods.

## Founding statement

This work was supported by DANONE GLOBAL RESEARCH & INNOVATION CENTER. The sponsor participated in the study design but had no access to the study data and no role in data analysis, interpretation of the results, or drawing of the conclusions.

## Declaration of competing interest

The authors declare the following financial interests/personal relationships which may be considered as potential competing interests:

Marta Bianchi reports financial support was provided by Danone Global Research & Innovation Center BV. If there are other authors, they declare that they have no known competing financial interests or personal relationships that could have appeared to influence the work reported in this paper.
