# Supplementary material for: Linking nutritional quality and climate impact of dairy and plant-based milk and yogurt alternatives: Evidence from three countries
Source: Curr Res Food Sci. 2026 Oct 13;13:101598. doi: 10.1016/j.crfs.2026.101598 (PMC13694937; doi:10.1016/j.crfs.2026.101598)
Supplement: Supplementary file 1 [file mmc1.docx]

# Supplementary

## 1. Complementing for missing data in the French food composition database

To fill gaps in nutrient content in the data obtained from the French CIQUAL(French Agency for Food, Environmental and Occupational Health & Safety), the following assumptions were made:

- Rice drink lacked protein content, which was taken from the US database (0,3 g/100g);
- Oat drink lacked protein content, which was set at 1 g/100g, calculated as average between oat drinks from the US and Swedish database.
- Chestnut drink lacked protein content, which was set to 0, according to the information available on the commercial product (provided by Danone).
- Coconut drink lacked protein content, which was taken from the US database (0,2 g/100g).
- Whole milk (pasteurized) lacked sugars content, and lactose content was therefore used (3,2 g/100g).
- A plant-based dessert without soybean lacked protein content, which was set at the same content as a similar item from the same database (1,6 g/100g).
- One of the soy drinks lacked saturated fat, which was set at the same content as a similar item from the same database (0,3 g/100g).
- Semi-skimmed milk with reduced lactose lacked a vast number of nutrients, which were therefore obtained from a semi-skimmed milk (regular lactose content) from the same database.
- A soy drink, with concentrated fruit juice, lacked a vast number of nutrients, which were therefore obtained from a similar item (unfortified sweet soy drink) from the same database.
- A yoghurt, sweteened with bifidus, lacked a vast number of nutrients, which were therefore obtained from a similar item (unfortified sweet soy drink) from the same database.
- Two yoghurt fat-free products containing NNS missing some nutrients were complemented.
- Similarly, among fruit-flavoured yoghurts, two items lacked vitamin D content which was taken from a similar product from the same database.
- In the case of other lacking nutrients which were expected to be negligible, content was set to zero.
- Where the nutrient content was reported below a threshold (i.e. level of analytical detection), the value was manually corrected to zero.

## 2. Dietary Refence Values

According to the methodology reviewed by the Nordic Nutrition Recommendations , the nutrients requirements used in this project were: recommended intake averages (RIA) for macronutrients (total fat, monounsaturated fat (MUFA), polyunsaturated fat (PUFA), linoleic acid (LA), alfa-linolenic acid (ALA), docosaexanoic acid (DHA), fibre); Recommended Intake (RI) (intake meeting the needs of 97.5% of the population) when available or Adequate Intake (AI) for minerals and vitamins where a RI is not established [5]; Maximum Recommended Intake (MRI) for total/added sugars, saturated fat and sodium.

Energy requirements were estimated assuming a moderate level of physical activity (PAL 1.6 MJ/day). By using country-specific reference body weights, the RI of protein was calculated as age and sex-specific g protein required per kg of body weight. For saturated fat and total/added sugars, the maximum recommended intake was calculated as a percentage of daily energy requirements. The fibre requirements were usually provided as an amount in grams per day or per MJ of energy.

For minerals such as iron and zinc, whose bioavailability is impacted by the amount of phytic acid, the RI was chosen referring to a diet with an average phytic acid intake of 600 mg/day.

Some further assumptions and methodological choices were made for the three countries and are reported in the following paragraphs.

French Nutrition Recommendations

For France, energy requirements used by the French ANSES (French Agency for Food, Environmental and Occupational Health & Safety) for children are based on EFSA (EFSA Panel on Dietetic Products, Nutrition and Allergies (NDA) 2013). For children aged 1-3 years, it is assumed a level of physical activity of 1.4 MJ/day, whereas for all other ages the energy requirements were calculated for a PAL of 1.6 MJ/day. Protein and fatty acids recommendations were obtained from AFSSA (French Agency of Food Safety). For MUFA and PUFA, the same reference intakes as in Sweden were used. Recommended fibre and total/added sugars intake was obtained from ANSES (Gaudichon et al. 2016). As the recommendation for dietary fibre intake in children was not provided, the amounts suggested by EFSA was used for ages 1-17 years (EFSA Panel on Dietetic Products, Nutrition, and Allergies (NDA) 2010).

French requirements for vitamins and minerals were obtained from ANSES (Anses - Agence Natl. Sécurité Sanit. L’alimentation L’environnement Trav. 2021), and refer to the latest update (2021).

The iron recommendation for the group of teenage girls (11-14 years) was set at 12 mg/day, calculated as the average of the RI for not menstruating and heavily menstruating girls. For the age group 15-17 years, the RI was assumed to be 13 mg/day, corresponding to the need of heavily menstruating girls.

For fluoride, the AI differed between males and females already in child age. For the groups 4-6, 7-10, 11-14 years the highest value between males and females was used.

Nordic Nutrition Recommendations

The Nordic Nutrition Recommendations do not give an indication for fibre intake in children [5], therefore the advice of 3.5 g/MJ given for adults was applied to children aged 1-10 years, with a maximum of 25 g/day. For fatty acids, the recommended intake ranges of LA, ALA, omega3s for adults were adopted also for children. As in the American recommendation, even the Nordic does not provide a specific intake for DHA or EPA, and therefore 250 mg/day of each compound was assumed as reasonable, in line with the French recommendations. For total MUFA and PUFA, the recommended intake was calculated as the average value in the recommended energy range (respectively 15 and 7,5 %E).

American Nutrition Recommendations

Recommended dietary intakes of fibre and protein were obtained from the Institute of Medicine (Trumbo et al. 2002). Indication of maximum daily intake of total/added sugars was obtained from the USDA (*Dietary Guidelines for Americans, 2020-2025*, n.d.). The Institute of Medicine provides age and sex-specific intake recommendations for LA and ALA, but not for EPA, DHA, or other LC omega-3s (‘Office of Dietary Supplements - Omega-3 Fatty Acids’, n.d.). However, the Dietary Guidelines for Americans suggest consuming 2-3 portions of fatty fish per week, which would provide approximately 250 mg DHA (*Dietary Guidelines for Americans, 2020-2025*, n.d.). Like France, even for the US, total recommended intake for MUFA and PUFA was aligned to the recommended %E as in Sweden.

Vitamins and minerals requirements for the US were obtained from the National Institutes of Health, Office of Dietary Supplements (‘Office of Dietary Supplements - Nutrient Recommendations and Databases’, n.d.) and refer to the following reports by the Institute of Medicine (*Dietary Reference Intakes for Thiamin, Riboflavin, Niacin, Vitamin B6, Folate, Vitamin B12, Pantothenic Acid, Biotin, and Choline* 1998; Institute of Medicine (US) Panel on Micronutrients 2001; Institute of Medicine (US) Standing Committee on the Scientific Evaluationof Dietary Reference Intakes 1997; Institute of Medicine (US) Panel on Dietary Antioxidants and Related Compounds 2000; *Dietary Reference Intakes for Water, Potassium, Sodium, Chloride, and Sulfate* 2005; Institute of Medicine (US) Committee to Review Dietary Reference Intakes for Vitamin D and Calcium 2011; Stallings et al. 2019).

**Table 1S.** Recommended intakes of nutrients included in NDS and DSI indicators.

| **Nutrients to favour** | **Nutrient index** | **DRV (France)** | **DRV (Sweden)** | **DRV (US)** | **Kind of DRV** |
| --- | --- | --- | --- | --- | --- |
| Protein (g) | NDS; DSI | 11-56 | 14-62 | 13-57 | AI, RI in US |
| Fiber (g) | NDS; DSI | 10-30 | 16-35 | 19-38 | AI |
| Linoleic acid (%E) | NDS | 4 | 2.5 | 7-17 (g/day) | RIA |
| Alpha linolenic acid (%E) | NDS | 1 | 0.5 | 0.7-1.6 (g/day) | RIA |
| DHA+EPA (mg) | NDS | 250 | 250 | 250 | RIA |
| Vitamin A (µg RE) | NDS | 250-750 | 300-800 | 300-900 | RI |
| Vitamin C (mg) | NDS | 20-110 | 25-110 | 15-90 | RI |
| Vitamin D (µg) | NDS; DSI | 15 | 10-20 | 15-20 | AI, RI in US |
| Vitamin E (mg) | NDS | 7-10 | 7-12 | 6-15 | AI, RI in US |
| Vitamin B1 (mg) | NDS | 0.4-1.2 | 0,5-1,3 | 0.5-1.2 | RI |
| Vitamin B2 (mg) | NDS; DSI | 0.6-1.6 | 0.6-1.6 | 0.5-1.3 | RI |
| Vitamin B3 (mg) | NDS | 7-19 | 7-19 | 6-16 | RI |
| Vitamin B6 (mg) | NDS; | 0.6-1.7 | 0.6-1.8 | 0.5-1.7 | RI |
| Folates (µg) | NDS; | 120-330 | 120-330 | 150-400 | RI |
| Vitamin B12 (µg) | NDS; DSI | 1.5-4 | 1.5-4 | 0.9-1.4 | AI |
| Calcium (mg) | NDS; DSI | 450-1150 | 450-1150 | 700-1300 | RI |
| Iron (mg) | NDS; DSI | 4-16 | 7-15 | 7-18 | RI |
| Potassium (mg) | NDS | 800-3500 | 850-3500 | 2000-3400 | AI |
| Magnesium (mg) | NDS | 180-380 | 170-350 | 80-420 | AI, RI in US |
| Iodine (µg) | NDS | 90-150 | 100-150 |  | AI |
| Selenium (µg) | NDS | 15-70 | 20-90 | 20-55 | AI, RI in US |
| Copper (mg) | NDS | 0.8-1.9 | n.a. | 0.34-0.9 | AI, RI in US |
| Zinc (mg) | NDS; DSI | 4.3-14 | 4.5-14 | 3-11 | RI |
| **Nutrients to limit** |  | **MRV (France)** | **MRV (Sweden)** | **MRV (US)** | **Kind of DRV** |
| SFA (%E) | LIM | 12 | 10 | 10 | MRI |
| Sodium (mg) | LIM | 1200-2300 | 1100-2300 | 1200-2300 | MRI |
| Added sugar (%E) | LIM | 10 | 10 | 10 | MRI |

RI: recommended intake; AI: adequate intake; RIA: recommended intake averages; MRI: Maximum recommended intake.

## 3. Demographic data for France, Sweden and the U.S.

**Table 2S.** Inhabitants in France, Sweden, and US for the year 2024 (INSEE 2025; Statistics Sweden 2025; US Census Bureau 2025)

| **France** | | | |
| --- | --- | --- | --- |
| Age group | Males | Females | Both |
| 1-3 yrs |  |  | 2027089 |
| 4-6 yrs |  |  | 2195918 |
| 7-10 yrs |  |  | 3182358 |
| 11-14 yrs |  |  | 3422019 |
| 15-17 yrs | 1345566 | 1263954 |  |
| 18-70 yrs | 21778380 | 22547304 |  |
| Tot | 23123946 | 23811258 | 57762588 |
| **Sweden** | | | |
| Age group | Males | Females | Both |
| 1-3 yrs |  |  | 324644 |
| 4-6 yrs |  |  | 354172 |
| 7-10 yrs |  |  | 494802 |
| 11-14 yrs | 260200 | 245578 |  |
| 15-17 yrs | 194618 | 182779 |  |
| 18-24 yrs | 434860 | 406889 |  |
| 25-50 yrs | 1830043 | 1740624 |  |
| 50-70 yrs | 1306711 | 1293440 |  |
| Tot | 4026432 | 3869310 | 9069360 |
| **US** | | | |
| Age group | Males | Females | Both |
| 1-3 yrs |  |  | 11159965 |
| 4-6 yrs |  |  | 11669435 |
| 9-13 yrs | 10644876 | 10156808 |  |
| 14-18 yrs | 11367564 | 10823533 |  |
| 19-30 yrs | 27485689 | 26513475 |  |
| 31-50 yrs | 44779638 | 44262399 |  |
| 51-70 yrs | 39625051 | 41650635 |  |
| Tot | 133902818 | 133406850 | 290139068 |

## 4. Carbon footprints – matching to food items

**Table 3S**. of the matching between processes of climate footprints from Agribalyse with products from the French, Swedish and American food databases.

| **Process** | **kg CO2-eq / kg product** | **Product description of products that have been matched to each process** |
| --- | --- | --- |
| Yogurt, fermented milk or dairy specialty, with chocolate shavings, with cream, with sugar, at plant {FR} S | 5,07 | Yogurt, fermented milk or dairy specialty, with chocolate shavings, with cream, with sugar |
| Yogurt, fermented milk or dairy specialty, w fruits, with sugar, at plant {FR} S | 1,72 | Fruit yogurt, different fat content |
| Yogurt, fermented milk or dairy specialty, plain, w sugar, at plant {FR} S | 1,11 | Yoghurt with added sugar |
| Yogurt, fermented milk or dairy specialty, plain, w cream, at plant {FR} S | 1,72 | Plain yogurt/fermented milk, with more than 3,6% fat |
| Yogurt, fermented milk or dairy specialty, plain, at plant {FR} S | 1,70 | Plain yogurt/fermented milk, with 3,6% fat or less |
| Yogurt, fermented milk or dairy specialty, flavoured, with sugar, at plant {FR} S | 1,05 | Flavoured yogurt (e.g. vanilla flavour) |
| Skimmed milk, at plant {FR} S | 0,84 | Milk around 0,5% fat |
| Semi-skimmed milk, at plant {FR} S | 0,96 | Milk around 1,5% fat |
| Milk, whole, pasteurised, at plant {FR} S | 1,14 | Milk with at least 3% fat |
| Dairy drink or fermented milk or yogurt, with fruits, with sugar, at plant {FR} S | 1,19 | Dairy drink or fermented milk or yogurt, with fruits, with sugar |
| Cow milk, consumption mix {FR} S | 1,13 | Fermented milk drink, plain, whole milk |
| Almond beverage, at plant {FR} S | 0,15 | Almond PBDA |
| Coconut drink, plain, at plant {FR} S | 0,62 | Coconut PBDA |
| Oat-based drink, plain, at plant {FR} S | 0,31 | Oat-based PBDA |
| Soy dessert, plain, refrigerated, at plant {FR} S | 0,38 | Soy dessert, plain |
| Soy dessert, w fruits, refrigerated, at plant {FR} S | 0,41 | Flavoured soy dessert and sweetened soy yogurt |
| Soy drink, flavoured, with sugar, at plant {FR} S | 0,21 | Soy milk, sweetened or flavoured |
| Soy drink, plain, at plant {FR} S | 0,21 | Soy milk, unsweetened |
| Soy drink, plain, fortified with calcium, at plant {FR} S | 0,21 | Fortified soy drink |
| Soy drink, w concentrated fruit juice, at plant {FR} S | 0,21 | Soy drink, w concentrated fruit juice |
| Chestnut-based drink, plain, prepacked (CIQUAL ID 18906) | 0,48 | Chestnut-based drink, plain |
| Rice-based drink, plain, prepacked (CIQUAL ID 18904) | 0,59 | Rice-based drink, plain |

## 5. Nutrient quality across dairy and PBDA categories according to NR-FI_milk_ and NR-FI_milk+PBD_

**Figure 1S.** Population-weighted median values of nutrient quality in French, Swedish and U.S. products according to two indicators. Data for 100 Kcal. Overall differences between groups were assessed using the Kruskal-Wallis test. Significant pairwise differences from Bonferroni-adjusted Wilcoxon rank-sum tests are indicated by horizontal lines and corresponding p-values. Bold lines indicate nutritionally relevant significant differences. Results were considered statistically significant at p≤0,05.

## 6. Nutrient quality across dairy and PBDA categories per 100 g

**Figure 2S.** Population-weighted median values of nutrient quality in French, Swedish and U.S. products according to two indicators. Data for 100 g. Overall differences between groups were assessed using the Kruskal-Wallis test. Significant pairwise differences from Bonferroni-adjusted Wilcoxon rank-sum tests are indicated by horizontal lines and corresponding p-values. Bold lines indicate nutritionally relevant significant differences. Results were considered statistically significant at p≤0,05.

## 7. Nutritional quality across dairy and PBDA categories when accounting for nutrients to limit

**Figure 3S.** Population-weighted median values of nutrient quality in French, Swedish and U.S. products according to NDS-LIM and DSI-LIM. Data for 100 g. Overall differences between groups were assessed using the Kruskal-Wallis test. Significant pairwise differences from Bonferroni-adjusted Wilcoxon rank-sum tests are indicated by horizontal lines and corresponding p-values. Bold lines indicate nutritionally relevant significant differences. Results were considered statistically significant at p≤0,05.

## 8. Nutritional quality across dairy and PBDA categories when capping for nutrients exceeding the DRVs

**Figure 4S.** Population-weighted median values of nutrient quality in French, Swedish and U.S. products according to two indicators and with application of capping for nutrients exceeding their DRVs. Data for 100 kcal. Overall differences between groups were assessed using the Kruskal-Wallis test. Significant pairwise differences from Bonferroni-adjusted Wilcoxon rank-sum tests are indicated by horizontal lines and corresponding p-values. Bold lines indicate nutritionally relevant significant differences. Results were considered statistically significant at p≤0,05.

## 9. Nutritional quality in fortified and unfortified dairy and PBDA categories

**Figure 5S.** Mean values of nutritional quality according to NDS and DSI in fortified and not-fortified French dairy and PBDA, per 100 kcal. Differences between fortified and not fortified were assessed using stratified Wilcoxon rank sum tests with Bonferroni correction for multiple testing.

**Figure 6S.** Mean values of nutritional quality according to NDS and DSI in fortified and not-fortified Swedish dairy and PBDA, per 100 kcal. Differences between fortified and not fortified were assessed using stratified Wilcoxon rank sum tests with Bonferroni correction for multiple testing.

**Figure 7S.** Mean values of nutritional quality according to NDS and DSI in fortified and not-fortified U.S. dairy and PBDA, per 100 kcal. Differences between fortified and not fortified were assessed using stratified Wilcoxon rank sum tests with Bonferroni correction for multiple testing.

Unfortfied sweetened or flavoured PBMAs (n=6) had higher quality than fortified (n=2) as they included soy-based and almon-based beverages with naturally high nutrient content, higher than the two oat milks that were fortified.

## 10. Ranking according to nutritional quality of French, Swedish and U.S. dairy and PBDA vs. reference foods

**Figure 8S.** Ranking of nutritional quality (highest to lowest) according to NDS and DSI in dairy, PBDA and 20 foods from other groups. Dark grey bars are data for France a), Sweden b) and the US c) based on population weighted averages. Light grey bars are population-weighted nutrient quality from single foods from the Swedish Food Composition database. All data is expressed as nutrient quality per 100 kcal of products ready for consumption.

## 11. Limiting nutrients (LIM) in dairy, PBDAs and reference foods

**Figure 9S.** Ranking of nutritional quality (highest to lowest) according to LIM (saturated fat, total sugars, sodium) in dairy, PBDAs and 20 foods from other groups. Dark grey bars are data for France a), Sweden b) and the US c) based on population weighted averages. Light grey bars are population-weighted nutrient quality from single foods from the Swedish Food Composition database. All data is expressed as nutrient quality per 100 g of products ready for consumption.

## 12. Carbon footprints of dairy and PBDAs

**Figure 10S**. Climate impact for dairy and PBDAs, per 1 kg of cooked/ready-to-eat food product at factory gate.

## 13. Carbon footprints per nutritional quality

**Figure11S**. Climate impact for dairy and PBDA products from France and nutrient quality assessed by the Nutrient Density Score. Median values per 100 kcal of cooked/ready to eat food product at factory gate.

# References

Anses - Agence Nationale de Sécurité Sanitaire de l’alimentation, de l’environnement et Du Travail. 2021. ‘Dietary Reference Values for Vitamins and Minerals’. May 7. https://www.anses.fr/en/content/dietary-reference-values-vitamins-and-minerals.

*Dietary Guidelines for Americans, 2020-2025*. n.d.

*Dietary Reference Intakes for Thiamin, Riboflavin, Niacin, Vitamin B6, Folate, Vitamin B12, Pantothenic Acid, Biotin, and Choline*. 1998. National Academies Press. https://doi.org/10.17226/6015.

*Dietary Reference Intakes for Water, Potassium, Sodium, Chloride, and Sulfate*. 2005. National Academies Press. https://doi.org/10.17226/10925.

EFSA Panel on Dietetic Products, Nutrition, and Allergies (NDA). 2010. ‘Scientific Opinion on Dietary Reference Values for Carbohydrates and Dietary Fibre’. *EFSA Journal* 8 (3): 1462. https://doi.org/10.2903/j.efsa.2010.1462.

EFSA Panel on Dietetic Products, Nutrition and Allergies (NDA). 2013. ‘Scientific Opinion on Dietary Reference Values for Energy’. *EFSA Journal* 11 (1): 3005. https://doi.org/10.2903/j.efsa.2013.3005.

French Agency for Food, Environmental and Occupational Health & Safety. n.d. ‘Ciqual- the French Food Composition Tables’. Accessed 25 July 2025. https://ciqual.anses.fr/.

Gaudichon, Claire C., Xavier Bigard, Jean-Michel Chardigny, et al. 2016. ‘Actualisation des repères du PNNS : élaboration des références nutritionnelles’. Report, Anses. https://anses.hal.science/anses-04314523.

INSEE. 2025. ‘Population Par Sexe et Groupe d’âges | Insee’. https://www.insee.fr/fr/statistiques/2381474.

Institute of Medicine (US) Committee to Review Dietary Reference Intakes for Vitamin D and Calcium. 2011. *Dietary Reference Intakes for Calcium and Vitamin D*. Edited by A. Catharine Ross, Christine L. Taylor, Ann L. Yaktine, and Heather B. Del Valle. The National Academies Collection: Reports Funded by National Institutes of Health. National Academies Press (US). http://www.ncbi.nlm.nih.gov/books/NBK56070/.

Institute of Medicine (US) Panel on Dietary Antioxidants and Related Compounds. 2000. *Dietary Reference Intakes for Vitamin C, Vitamin E, Selenium, and Carotenoids*. National Academies Press (US). http://www.ncbi.nlm.nih.gov/books/NBK225483/.

Institute of Medicine (US) Panel on Micronutrients. 2001. *Dietary Reference Intakes for Vitamin A, Vitamin K, Arsenic, Boron, Chromium, Copper, Iodine, Iron, Manganese, Molybdenum, Nickel, Silicon, Vanadium, and Zinc*. National Academies Press (US). http://www.ncbi.nlm.nih.gov/books/NBK222310/.

Institute of Medicine (US) Standing Committee on the Scientific Evaluationof Dietary Reference Intakes. 1997. *Dietary Reference Intakes for Calcium, Phosphorus, Magnesium, Vitamin D, and Fluoride*. The National Academies Collection: Reports Funded by National Institutes of Health. National Academies Press (US). http://www.ncbi.nlm.nih.gov/books/NBK109825/.

‘Office of Dietary Supplements - Nutrient Recommendations and Databases’. n.d. Accessed 8 July 2025. https://ods.od.nih.gov/HealthInformation/nutrientrecommendations.aspx.

‘Office of Dietary Supplements - Omega-3 Fatty Acids’. n.d. Accessed 25 July 2025. https://ods.od.nih.gov/factsheets/Omega3FattyAcids-HealthProfessional/.

Stallings, Virginia A., Meghan Harrison, and Maria Oria, eds. 2019. *Dietary Reference Intakes for Sodium and Potassium*. With Committee to Review the Dietary Reference Intakes for Sodium and Potassium, Food and Nutrition Board, Health and Medicine Division, and National Academies of Sciences, Engineering, and Medicine. National Academies Press. https://doi.org/10.17226/25353.

Statistics Sweden. 2025. ‘Population by Age and Sex. Year 1860 - 2024’. Statistikdatabasen. https://www.statistikdatabasen.scb.se:443/pxweb/en/ssd/START__BE__BE0101__BE0101A/BefolkningR1860N/.

Trumbo, Paula, Sandra Schlicker, Allison A. Yates, Mary Poos, and Food and Nutrition Board of the Institute of Medicine, The National Academies. 2002. ‘Dietary Reference Intakes for Energy, Carbohydrate, Fiber, Fat, Fatty Acids, Cholesterol, Protein and Amino Acids’. *Journal of the American Dietetic Association* 102 (11): 1621–30. https://doi.org/10.1016/s0002-8223(02)90346-9.

US Census Bureau. 2025. ‘National Population by Characteristics: 2020-2024’. Census.Gov. https://www.census.gov/data/tables/time-series/demo/popest/2020s-national-detail.html.
